# Successful anaesthetic management of a COVID-positive patient with multiple comorbidities: regional anaesthesia to the rescue

**DOI:** 10.1186/s42077-021-00176-0

**Published:** 2021-09-20

**Authors:** Shweta Aghi, Srinivasan Muthuswamy, Nidhi Bhatia, Kamal Kajal, Anjishnujit Bandyopadhyay

**Affiliations:** Department of Anaesthesia and Intensive Care, Post Graduate Institute of Medical Education and Research, Chandigarh, India

**Keywords:** Popliteal nerve block, Wrist block, Regional anaesthesia, COVID-19 pneumonia

## Abstract

The perioperative anaesthetic management of a case of COVID-19 pneumonia with multiple systemic comorbidities, posted for unilateral below knee amputation and debridement of hand, poses a uniquely challenging scenario for the anaesthesiologist. We hereby report such a case which was managed successfully using ultrasound-guided popliteal and wrist block along with perioperative use of high flow nasal cannula, incentive spirometry and awake proning.

## Background

Emergency surgery in a patient with multiple systemic comorbidities is a difficult challenge for the perioperative physician. The presence of coronavirus disease 2019 (COVID-19) pneumonia only complicates the situation further. We hereby report the successful perioperative management of a 44-year-old COVID-19-positive patient with multiple comorbidities who underwent unilateral below knee amputation.

## Case description

A 44-year-old gentleman, known case of systemic scleroderma, secondary Sjogren’s disease, Interstitial lung disease, severe Raynaud’s disease and diabetes mellitus with poor compliance to therapy, was admitted to intensive care unit (ICU) after being diagnosed with severe COVID-19 pneumonia requiring invasive mechanical ventilation. With general supportive ICU care, he improved clinically and was extubated. Thereafter, he was managed using high flow nasal cannula (HFNC) with intermittent pressure support ventilation via non-invasive ventilation (NIV) mask.

During his stay in the ICU, he developed pericardial effusion and acute kidney injury (AKI). He received 3 sessions of intermittent haemodialysis in view of anuria, following which his urine output and other renal parameters improved. Subsequently, he developed dry gangrene of the right foot and right hand most probably as a complication of Raynaud’s disease, despite being on a prophylactic dose of low molecular weight heparin. He was posted for emergency foot debridement and toe amputation of the right lower limb and debridement of the lesion over the right hand. Pericardial effusion was drained with the placement of a 10 F fenestrated pigtail catheter on the day of the surgery.

Management of the case perioperatively posed a unique anaesthetic challenge owing to the myriad of comorbidities. Preoperatively, the patient was on HFNC FiO_2_ 0.5 at 40 l/min, respiratory rate of 30/min, SpO2 94–95% with a blood pressure of 110/64 mmHg and heart rate 94/min. General anaesthesia (GA) was considered high risk and hence avoided for the following reasons: firstly, to prevent the need for intubation, considering the increased risk of post-operative pulmonary complications in COVID-positive patients (Nepogodiev et al. 2020). Secondly, the patient had an anticipated difficult airway owing to his partial edentulous status, limited mouth opening and limited head extension. Lastly, avoiding GA would help avoid engaging in aerosol generating procedures for the safety of health care workers as the patient was still polymerase chain reaction (PCR) positive for COVID. Since the patient had received a dose of low weight molecular heparin within the last 12 h, neuraxial anaesthesia was rendered relatively unsafe (Horlocker et al. 2018).

Hence, we performed a successful ultrasound (USG) guided popliteal sciatic and saphenous nerve block for foot debridement and toe amputation of the right lower limb and wrist block by landmark technique for debridement of the right-hand lesion. Twenty millilitres of drug (10 ml of 0.5% plain bupivacaine + 10 ml of 2% lignocaine with adrenaline) was administered into the popliteal fossa while a total of 10 ml of drug (5 ml of 0.5% bupivacaine + 5 ml of 2% lignocaine with adrenaline) was used for the wrist block. A total of 15 ml of 0.5% plain bupivacaine and 15 ml of 2% lignocaine were used, both of which were well within the safety limits of toxicity. All health care workers in the operating room donned a fitted n95 mask with level 3 personal protective equipment. The patient maintained a saturation of 96–98% on venturi mask at an FiO_2_ of 0.6. HFNC was kept ready for use, in backup, in case the patient experienced respiratory distress or desaturation. The surgery was completed without any complications. Postoperatively, the patient was put on a multimodal analgesic regimen for round the clock pain relief. Chest physiotherapy, awake proning, incentive spirometry and ambulation were continued till the patient was discharged from ICU.

## Discussion

This case highlights how regional anaesthesia techniques are an indispensable tool in the armamentarium of the anaesthesiologist and can be utilised to facilitate surgery in high-risk patients as well as provide a valuable alternative to GA.

High-risk patients with multiple uncontrolled comorbidities are in themselves a challenge for the perioperative physician. The presence of COVID-19 adds an unprecedented aspect to this challenge. Each lesson learnt is a step forward in developing a standardised approach for difficult situations. Through this case, we have described our successful attempt at securing a safe and satisfactory outcome for such patients. Different factors such as the use of regional anaesthesia, perioperative use of HFNC and awake proning also seemed to have contributed to this success.

## Data Availability

Not applicable.

## Abbreviations

AKI: Acute kidney injury
COVID-19: Coronavirus disease 2019
GA: General anaesthesia
HFNC: High flow nasal cannula
ICU: Intensive care unit
NIV: Non-invasive ventilation
USG: Ultrasonography
